# Nanotextured silk fibroin/hydroxyapatite biomimetic bilayer tough structure regulated osteogenic/chondrogenic differentiation of mesenchymal stem cells for osteochondral repair

**DOI:** 10.1111/cpr.12917

**Published:** 2020-10-01

**Authors:** Lingling Shang, Baojin Ma, Fulei Wang, Jianhua Li, Song Shen, Xiaoyuan Li, Hong Liu, Shaohua Ge

**Affiliations:** ^1^ Department of Periodontology School and Hospital of Stomatology Cheeloo College of Medicine Shandong University & Shandong Key Laboratory of Oral Tissue Regeneration & Shandong Engineering Laboratory for Dental Materials and Oral Tissue Regeneration Jinan China; ^2^ State Key Laboratory of Crystal Materials Shandong University Jinan China

**Keywords:** biomimetic bilayer structure, hydroxyapatite nanowire, nanotexture, osteochondral repair, silk fibroin

## Abstract

**Objectives:**

Articular cartilage plays a vital role in bearing and buffering. Injured cartilage and subchondral bone repair is a crucial challenge in cartilage tissue engineering due to the peculiar structure of osteochondral unit and the requirement of osteogenic/chondrogenic bi‐directional differentiation. Based on the bionics principle, a nanotextured silk fibroin (SF)‐chondroitin sulphate (CS)/hydroxyapatite (HAp) nanowire tough bilayer structure was prepared for osteochondral repair.

**Methods:**

The SF‐CS/HAp membrane was constructed by alcohol‐induced β‐sheet formation serving as the physical crosslink. Its osteochondral repairing capacity was evaluated by culturing bone marrow mesenchymal stem cells (BMSCs) in vitro and constructing a rat osteochondral defect model in vivo.

**Results:**

The bilayer SF‐CS/HAp membrane with satisfactory mechanical properties similar to natural cartilage imitated the natural osteochondral unit structural layers and exerted the function of bearing and buffering timely after in vivo implantation. SF‐CS layer upregulated the expression of chondrogenesis‐related genes of BMSCs by surface nanotopography and sustained release CS. Meanwhile, nanotextured HAp layer assembled with nanowire endowed the membrane with an osteogenic differentiation tendency for BMSCs. In vivo results proved that the biomimetic bilayer structure dramatically promoted new cartilage formation and subchondral bone remodelling for osteochondral defect model after implantation.

**Conclusions:**

The SF‐CS/HAp biomimetic bilayer membrane provides a promising strategy for precise osteochondral repair.

## Graphical abstract

Nanotextured silk fibroin (SF)‐chondroitin sulphate (CS)/hydroxyapatite (HAp) nanowire membrane with biphasic structure and desired mechanical performances due to β‐sheet formation was prepared to enhance chondrogenic/osteogenic bi‐directional differentiation of bone marrow mesenchymal stem cells (BMSCs) in vitro and facilitate osteochondral repair in vivo.

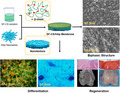

## 1 INTRODUCTION

Articular cartilage possesses the function of bearing and buffering and is an essential foundation for human body motion.^1^ The peculiar structure of cartilage tissue, including deficiency of blood vessels and nerve, few chondrocytes and low proliferation ability, determines the poor self‐repair capability after injury. Clinically, treatment of articular cartilage injury has always been an intractable problem owing to the weak self‐repair ability of cartilage.^2^, ^3^ Currently, various cartilage repairing materials have been prepared from a variety of natural macromolecular compounds, such as gelatin, collagen, hyaluronic acid and chitosan.^4^, ^5^, ^6^, ^7^, ^8^, ^9^, ^10^ It is worth noting that cartilage defect was usually companied by subchondral bone injury. Thus, the hierarchical scaffolds imitating the structure and function of cartilage and subchondral bone have been designed for the bionic repair of the integrated osteochondral unit.^11^, ^12^, ^13^ Some hierarchical materials have achieved excellent efficacies in the restoration of osteochondral defect, but certain limitations existed, especially in mechanical performance. Once implanted into the arthrosis, many materials had weak mechanical strength to support heavy loads and stress in time. Therefore, besides biocompatibility and biological effects, suitable mechanical performance is also critical for osteochondral repair.^2^ Furthermore, the hierarchical scaffolds, which used one material loaded with various chondrogenic and osteogenic inducers in two layers, could not truly simulate osteochondral repair environment. A few studies have developed biphasic scaffolds using different materials, such as the electrospinning silk/bioactive glass composite scaffolds and graphene‐polycaprolactone (PCL)/bioactive glass scaffolds, to mimic the hierarchical complexity of the osteochondral interface.^14^, ^15^ However, owing to the absence of continuous phases in different layers, the connectivity between the two layers was insufficient. These limitations become a hindrance to clinical transformation. Accordingly, it is essential to develop a seamless bilayer structure with preferable mechanical property to achieve precise osteochondral regeneration.

Hydroxyapatite (HAp) has the superiority of structural and functional similarity to mineral composition of nature bones.^16^ Our previous study demonstrated that one‐dimensional HAp short nanowires had admirable osteogenic potential by nanostructure stimulation without any growth factors assistance.^17^ Therefore, HAp short nanowires are suitable as osteoinduction layer for repairing subchondral bone in the biphasic structure. Recently, Silk fibroin (SF)‐based materials are emerging as an excellent matrix for cartilage tissue engineering.^18^, ^19^, ^20^ The 3D printed SF‐glycidyl‐methacrylate hydrogel enhanced chondrogenic differentiation of encapsulated cells and the formation of new cartilage‐like tissue.^21^ However, many SF‐based scaffolds were short of desired mechanical properties and unable to bear and buffer timely. Even after crosslinking, elastic modulus of most hydrogels is only about 0.01‐0.2 MPa,^22^, ^23^, ^24^, ^25^, ^26^ which is far from natural cartilage tissue (~4.1 MPa).^27^, ^28^ Chondroitin sulphate (CS), a main glycosaminoglycan in cartilage extracellular matrix (ECM), is extensively used for treating osteoarthritis and cartilage tissue engineering.^29^ Besides intrinsic biological activities, physical cues, such as surface nanotopography and mechanical properties of materials, have synergistic effects on committed differentiation and tissue regeneration since the physical microenvironment for cell survival had tissue‐specific topography and rigidity.^30^, ^31^

In this study, we prepared an SF‐CS/HAp bilayer membrane following a biomimetic microstructure engineering design principle. The physicochemical properties, releasing profile and cell compatibility were characterized. Subsequently, both in vitro and in vivo studies were implemented to assess the effectiveness of the proposed design principle.

## 2 MATERIALS AND METHODS

### 2.1 Preparation of SF‐CS/HAp membrane

HAp short nanowires were synthesized according to our previous method.^17^ Degumming process was in accordance with a previous study.^32^ Degumming SF and CS were added into formic acid solution containing CaCl_2_ to form SF‐CS solution. HAp short nanowires dispersed in ethanol were poured into a compatible mould, and HAp film was formed after dry. The SF‐CS solution was poured to HAp film. After the solvent evaporation, SF‐CS/HAp composite was immersed into deionized water to remove CaCl_2_, followed by solidifying with ethanol.

### 2.2 Physicochemical characterization of SF‐CS/HAp membrane

The morphology and structure characteristics were observed under an S‐4800 scanning electron microscope (SEM) (Hitachi) and a JEM‐2100 transmission electron microscope (TEM) (Jeol). To analyse the crystallinity, X‐ray diffractograms (XRD) were performed on a Bruker D8 advance powder diffractometer equipped with a Cu Kα sealed tube. Chemical composition was assessed by a fourier transform infrared spectroscopy (Thermo Scientific). The tensile mechanical properties of the membrane (8 × 10 × 0.4 mm^3^) were measured by a universal testing machine (Instron 3340). All samples were stretched at a speed of 0.437 mm/s. Nanoindentation measurements were performed using a three‐sided pyramidal Berkovich diamond indenter with a nominal edge radius of 20 nm (faces 65.3° from vertical axis) attached to a fully calibrated nanoindenter (Nano Indenter G200, Agilent). The indentations were conducted with a continuous stiffness measurement (CSM) module and a speed of 10 nm/s. Topographic features and roughness of SF‐CS surfaces were studied under an atomic force microscope (AFM; Dimension Icon, Bruker). The root mean square average (Ra) roughness was calculated from 0.5 × 0.5 μm^2^ image areas.

### 2.3 CS release from SF‐CS/HAp membrane

SF‐CS/HAp membrane was soaked into 2 mL phosphate‐buffered saline (PBS, HyClone) in a centrifuge tube and then placed in a constant temperature oscillator (37°C, 100 rpm). At the desired time intervals (3, 5, 7, 9, 11, 13, 17 days), 2 mL PBS was extracted and equivalent fresh PBS was replenished to the tube. The absorbance of the collected PBS was measured at 260 nm wavelength using an ultraviolet spectrophotometry. The release profile was obtained by calculating the concentrations of CS according to the standard curve.

### 2.4 Cell culture and cytocompatibility evaluation of SF‐CS films

Bone marrow mesenchymal stem cells were harvested from healthy male SD rats (3‐4 weeks) by direct panmyeloid adherence. The study protocol was approved by the Medical Ethical Committee of School of Stomatology, Shandong University (Protocol Number: GD201801). BMSCs were cultured with α‐minimum essential medium (α‐MEM, HyClone) supplemented with 10% foetal bovine serum (FBS, BioInd) and 1% antibiotics at 37°C in a 5% CO_2_ incubator. BMSCs were seeded on SF‐CS membranes and incubated for 48 hours. Cells were collected by trypsinization and seeded into a new culture plate in case of adsorbing the formazan dye by SF. Afterwards, cell counting kit‐8 (CCK8, Dojindo Laboratories) solution was added. The optical absorbance value was measured at 450 nm wavelength using a microplate reader (SPECTROstar Nano, BMG Labtech).

### 2.5 Osteogenic immunofluorescence staining

After cultured for 14 days, BMSCs on different sides of the membrane were collected by trypsinization and seeded into a new culture plate. Cells were then fixed with 4% paraformaldehyde and permeabilized using 0.1% Triton X‐100 (Solarbio). After blocking with 10% normal goat serum, cells were incubated with an anti‐osteocalcin (OCN) primary antibody (Abcam) and an anti‐osteopontin (OPN) primary antibody (Abcam) at 4°C overnight. Cy3‐conjugated goat anti‐rabbit and Alexa Fluor 488‐conjugated goat anti‐mouse IgG secondary antibody were used for OPN and OCN staining in the dark for 1 hour, respectively. Images were observed under a fluorescence microscope (OLYMPUS IX73).

### 2.6 RNA isolation and quantitative Real‐Time Polymerase Chain Reaction (qRT‐PCR)

Respectively, BMSCs were seeded onto SF‐CS sides with chondroinductive medium (CM) and HAp sides with osteoinductive medium (OM) for 14 days. Total RNA of the cells was isolated using TRIzol reagent. RNA was reverse‐transcribed to complementary DNA, and qRT‐PCR was performed with LightCycler 96 Real‐Time PCR System (Roche) using SYBR^®^ Premix Ex Taq™ II (Takara) to detect the gene levels of OCN, OPN, collagen type II alpha 1 (COL2A1), Aggrecan (ACAN) and sex‐determining region Y box protein 9 (SOX‐9). The relative transcript levels of the target gene expressions were normalized to β‐actin and expressed as mean ± SD (n = 3).

### 2.7 Alcian blue staining

Bone marrow mesenchymal stem cells were seeded onto SF sides containing 4%, 8%, 12% and 16% CS in CM for 14 days. Cells were washed with PBS thrice and fixed with 4% paraformaldehyde. Subsequently, cells were stained with Alcian blue staining solution (Solarbio) and the nuclei were counterstained with redyeing solution. Images were obtained using a microscope (OLYMPUS BX53).

### 2.8 Animal osteochondral defect models

Animal experiments were approved by the Medical Ethical Committee of School of Stomatology, Shandong University (Protocol Number: GD201801) and carried out in accordance with the National Institutes of Health Guide for the Care and Use of Laboratory Animals (NIH Publications No. 8023, revised 1978). Eight‐week‐old male SD rats were used in this study. A cylindrical osteochondral defect (with 1.5 mm diameter and 1 mm depth) was created with a dental drill at the trochlear grooves of the distal femurs. The rats were randomly divided into five groups, and different membranes were implanted into the defects: (a) negative control (NC); (b) SF membrane; (c) SF/HAp membrane; (d) SF‐CS membrane; (e) SF‐CS/HAp membrane. At 6 and 12 weeks postoperatively, the rats were sacrificed by excessive pentobarbital anaesthesia and fixed with 4% paraformaldehyde by cardiac perfusion. The distal femurs of the rats were harvested for the following experiments.

### 2.9 Gross observation and micro‐computational tomography (micro‐CT) analysis

Images of gross specimen were captured using a Digital SLR cameras (Canon). The effect of cartilage rehabilitation was evaluated by three blinded independent researchers according to ICRS histological scoring standard. To analyse the remodelling of subchondral bone, a micro‐CT (Perkin Elmer) was applied to scan the specimens with scan settings of voltage 90 kV, current 88 μA and voxel resolution 50 μm at 360°. CT vox and CT analysis software were used to reconstruct the images for 3D visualization and analysis. Quantitatively, several indexes including the percentage of bone volume (bone volume/tissue volume, BV/TV), trabecular thickness (Tb.Th), trabecular numbers (Tb.N) and trabecular separation (Tb. Sp) were measured and calculated for the assessment of bone regeneration.

### 2.10 Histological analysis

H&E staining, toluidine blue staining, Saf‐O staining and immunohistochemical staining with polyclonal rabbit anti‐Col‐II antibody and anti‐Col‐I antibody (Abcam) were performed to appraise cartilage regeneration in line with the manufacturer's protocols. The specimens were observed under a BX53 microscope. The cartilage regeneration at week 6 and 12 was assessed by histological scoring system for evaluation of cartilage repair.^33^ The immunohistochemical staining intensity for Col‐II and Col‐I was calculated by Image‐Pro Plus 6.0 software.

### 2.11 Statistical analysis

Data were presented as mean ± standard deviation (SD). Differences between more than two experimental groups and NC group were analysed by one‐way ANOVA followed by Tukey's HSD comparison test, and variance between two groups was compared by two‐way *t* test with GraphPad Prism software (version 6, by MacKiev Software). *P* < .05 was considered statistically significant.

## 3 RESULTS

### 3.1 Characterization of SF‐CS/HAp membrane

**Figure 1:**
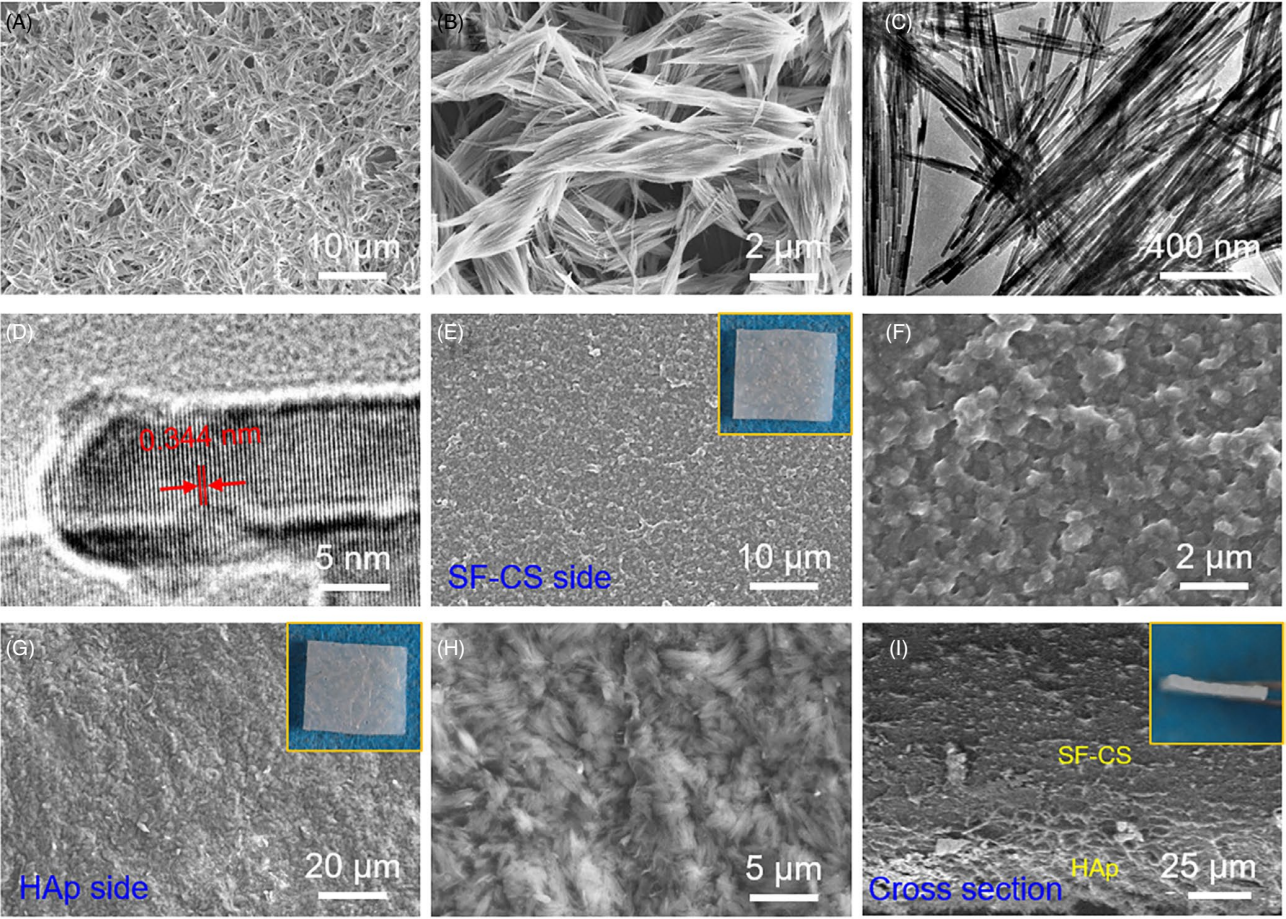
Morphology characterization of SF‐CS/HAp membrane. A and B, SEM images of HAp short nanowires under different magnifications. C, TEM images of HAp short nanowires. D, HRTEM images of HAp short nanowires. E and F, SEM images of SF sides under different magnifications and the corresponding physical picture. G and H, SEM images of HAp sides under different magnifications and the corresponding physical picture. I, Sectional view of SF‐CS/HAp membrane and the corresponding physical picture

The morphology of HAp nanowires and SF‐CS/HAp membrane was characterized by SEM and TEM (Figure 1). The HAp short nanowires with approximate length of 2 μm and width of 10 nm were intertwined and twisted with each other (Figure 1A,B). HRTEM characterization was performed to observe crystal lattice and growth direction of the nanowires (Figure 1C,D). HAp nanowires possessed typical lattice spacing at (002) (~0.344 nm), indicating that the nanowires grew along the *c* axis.^17^ SF membrane was translucent and had good flexibility (Figure S1A). The incorporation of CS and HAp partly reduced the transparency (insert of Figure 1E,G,I). SF surface of the bilayer structure had irregular nanotopography (Figure 1F), and HAp side maintained the original morphology of short nanowires as mentioned above (Figure 1H). In addition, the inseparable combination of SF‐CS and HAp realized a seamless interface between the organic and inorganic phases, which was based on the permeation of glutinous SF‐CS solution to HAp nanowires. Further, elements distribution of SF‐CS/HAp membrane cross‐section demonstrated that two layers were continuous and gradual transition (Figure S1B,C).

**Figure 2:**
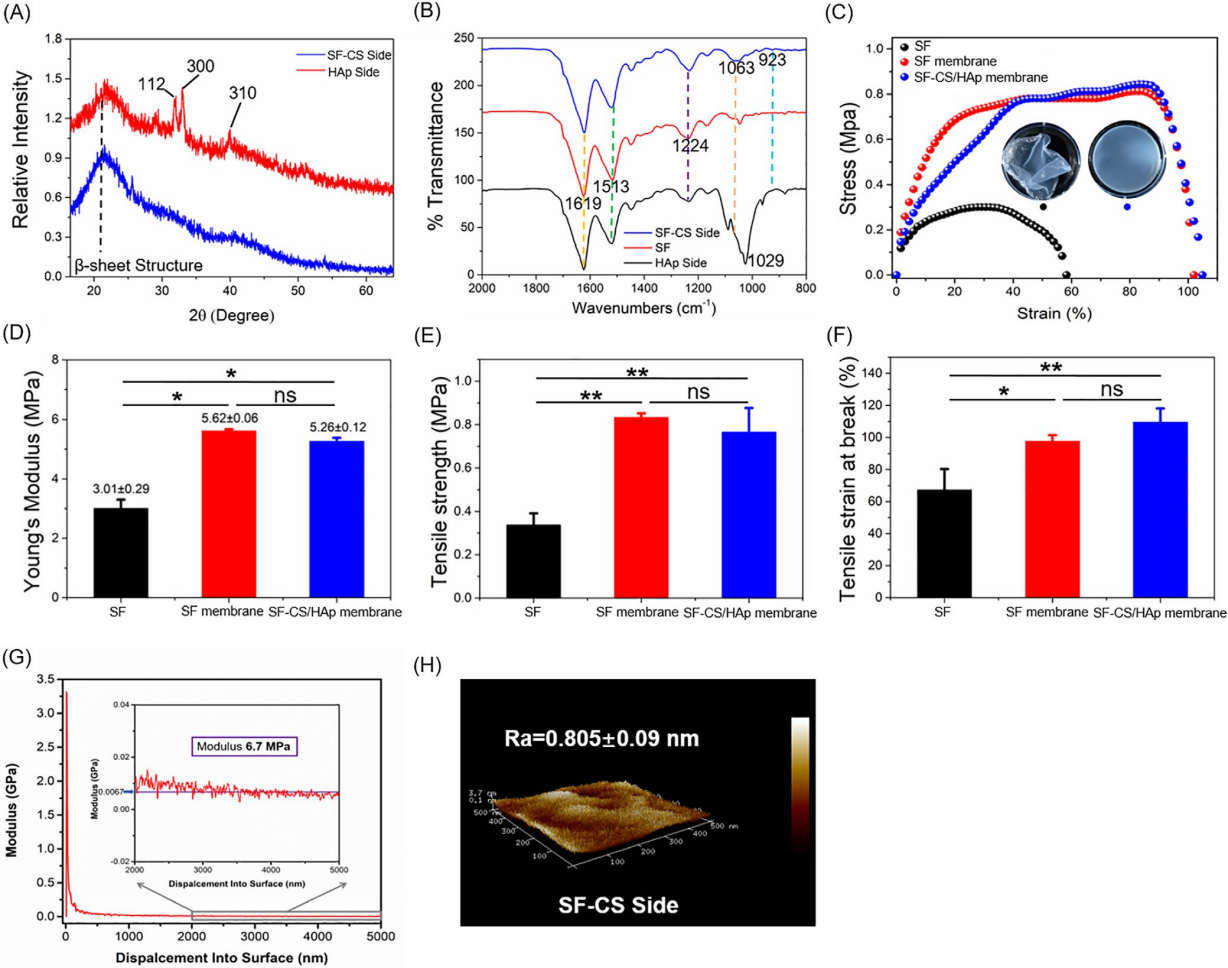
Structure and mechanical property characterization of SF‐CS/HAp membrane. A, XRD patterns of SF‐CS side and HAp side of SF‐CS/HAp membrane. B, FTIR spectra of SF membrane, HAp side of SF‐CS/HAp membrane, SF‐CS side of SF‐CS/HAp membrane. C, Tensile stress‐strain curves of SF (without ethanol treatment), SF membrane and SF‐CS/HAp membrane. Insert is the physical picture of SF (without ethanol treatment) and SF membrane. D, Young's modulus of different membranes. E, Tensile strength of different membranes. F, Tensile strain at break of different membranes. G, Compressive modulus of SF‐CS/HAp membrane by nanoindentation analyses. H, 3D AFM image for SF‐CS side of SF‐CS/HAp membrane. *P < .05, **P < .01, ns, no statistical significance

XRD patterns and FTIR spectra were obtained to confirm the crystal phase and chemical properties. Both HAp nanowires (Figure S2A) and HAp side of SF‐CS/HAp membrane (Figure 2A) had the typical diffraction peaks of HAp (Powder Diffraction File no. 74‐0565, Joint Committee on Powder Diffraction Standards, 2009). Additionally, there was a broad peak located at 2θ = 24° in the SF‐CS/HAp membrane (Figure 2A). FTIR spectra of HAp nanowires (Figure S2B) and HAp side of SF‐CS/HAp membrane (Figure 2B) had the characteristic peak of HAp at 1029 cm^−1^ (‐PO_4_^3−^). SF membrane, HAp side and SF‐CS side of SF‐CS/HAp membrane showed the typical peaks of silk at 1513 and 1619 cm^−1^, which belonged to amide II and I vibration, respectively (Figure 2B). The peaks at 1224, 1063 and 923 cm^−1^ were observed in FTIR spectra for SF‐CS side of SF‐CS/HAp membrane (Figure 2B).

Representative tensile strain‐stress curves were shown in Figure 2C, and the area under the tensile strain‐stress curves represented the toughness of membranes. SF without ethanol treatment (SF) was flabby (insert of Figure 2C), while SF membrane and SF‐CS/HAp membrane (ethanol‐treated) possessed high and similar toughness due to β‐sheet structure formation. After ethanol treatment, Young's modulus of SF membrane (~5.6 MPa) and SF‐CS/HAp membrane (~5.26 MPa) significantly increased (Figure 2D, *P* < .05), and the tensile strength (tensile stress at break) of SF membrane and SF‐CS/HAp membrane reached up to ~0.83 and ~0.77 MPa, respectively (Figure 2E). The tensile strain was ~67% when SF without ethanol treatment broken (Figure 2F), whereas SF membrane and SF‐CS/HAp membrane stretched by ~97% and ~110%, indicating that ethanol solidification significantly enhanced the stretchability of SF. SF‐CS/HAp membrane still maintained the integrity and generated small deformation under tensile stress of the weight (Figure S1D). The compressive modulus of SF‐CS/HAp membrane was ~6.7 MPa (Figure 2G), and representative compression stress‐strain curve presented good self‐recovery property of SF‐CS/HAp membrane (Figure S3). Three‐dimensional (3D) representative AFM image presented the topographical features of the SF‐CS side of SF‐CS/HAp membrane (Figure 2H). Surface of SF‐CS/HAp membrane was relatively flat, and Ra value of the surface was 0.805 ± 0.09 nm, indicating that surface of SF‐CS/HAp membrane was conducive to decrease the friction and wear after implantation.

Furthermore, ultraviolet spectrophotometry was used to evaluate the controlled release capacity of the membranes. CS was continuously released from SF‐CS/HAp membranes immersed in PBS, with an initial burst release (~20%) at the first 3 days from the surface, and the releasing reached a balance at day 11 (Figure S4).

### 3.2 Chondrogenic and osteogenic differentiation potentials of SF‐CS/HAp membrane

#### 3.2.1 Cell viability

**Figure 3:**
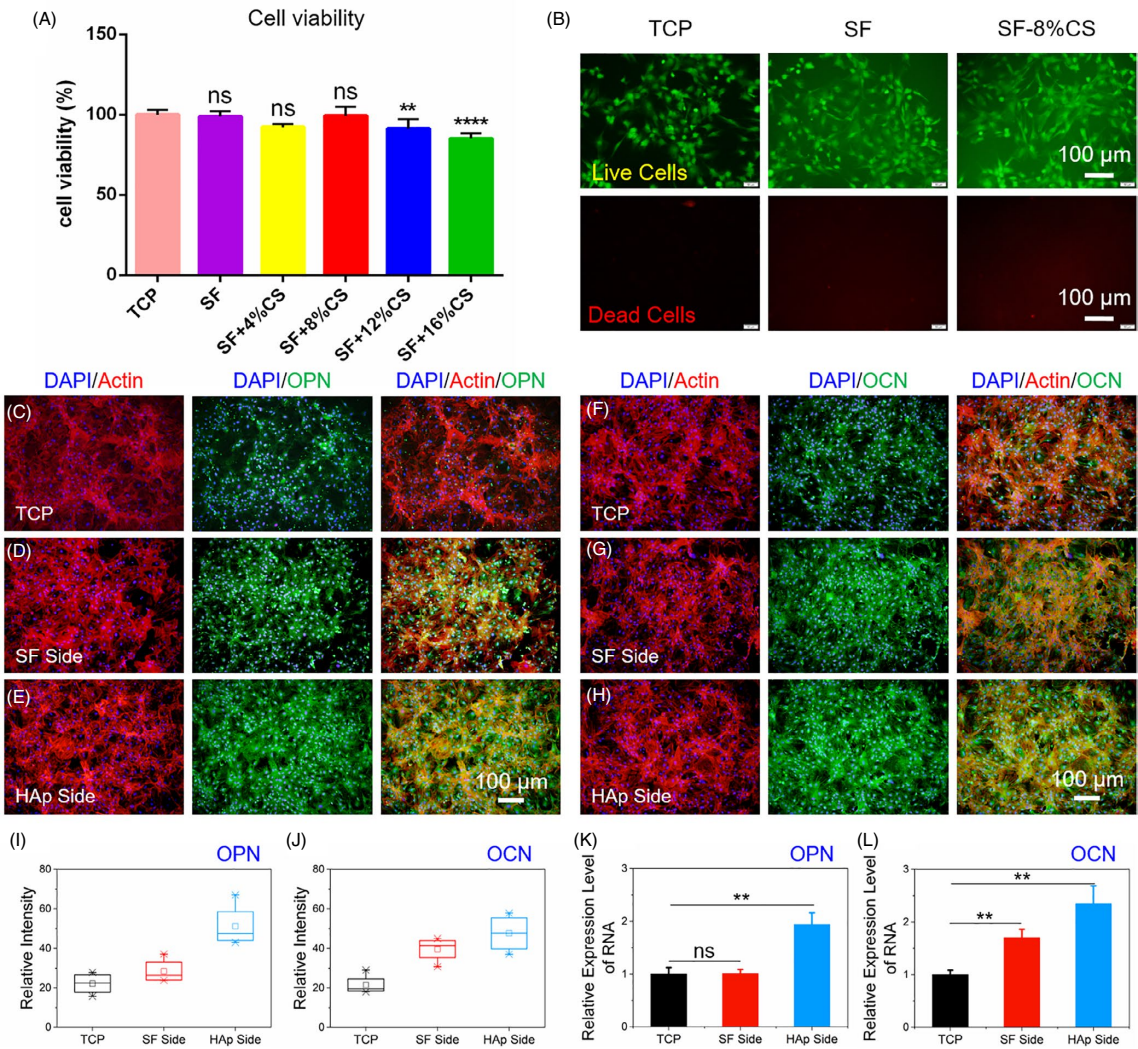
The cytocompatibility evaluation and osteogenic differentiation of BMSCs. A, Cell vitality detected by CCK‐8 assay after cultivation on TCP and SF membrane with various concentrations of CS for 48 h. B, Live/dead cell staining on TCP, SF and SF‐CS (8%) membranes after cultivation for 48 h. Live cells (green), Dead cells (red). C‐E, OPN staining and cytoskeletal staining of BMSCs on TCP, HAp and SF sides. F‐H, OCN staining and cytoskeletal staining of BMSCs on TCP, HAp and SF sides. I and J, Quantitative analysis of OPN and OCN fluorescence intensity. K and L, qRT‐PCR analysis of the relative mRNA levels of OCN and OPN after cultured for 14 d. **P < .01 and ****P < .0001, compared with TCP, ns, no statistical significance compared with TCP

Pure SF, 4% and 8% CS‐loaded SF did not affect BMSCs proliferation after incubation for 48 hours. Although 12% and 16% CS inhibited proliferation (*P* < .01), BMSCs still kept relatively high viability (Figure 3A). To observe cell status visually, BMSCs were incubated for 48 hours and stained with a Live/Dead kit (red indicates dead cells; green indicates live cells). Cells seeded on SF‐8% CS membrane exhibited high viability with a majority of live cells and rare dead cells, which was comparable with those cultured on TCP (Figure 3B).

#### 3.2.2 Osteogenic and chondrogenic differentiation capacity assessment

To evaluate osteogenic capacity, BMSCs were separately inoculated onto TCP and two sides of SF‐CS/HAp membrane within OM for 14 days. The protein and gene levels of OPN and OCN were assessed by immunofluorescent staining and qRT‐PCR, respectively. The cytoskeletal staining assay indicated that cell density on HAp side and SF side was roughly equivalent to that on TCP. Nevertheless, the protein level of OCN and OPN was significantly enhanced for BMSCs cultured on HAp side, compared with those on TCP and SF side (Figure 3C‐J). The result was further supported by qRT‐PCR which showed that the gene level of OPN and OCN on HAp side was 2.3‐ and 1.9‐folds as much as that on TCP (*P* < .01, Figure 3K,L). Therefore, HAp side facilitated osteogenic differentiation of BMSCs.

**Figure 4:**
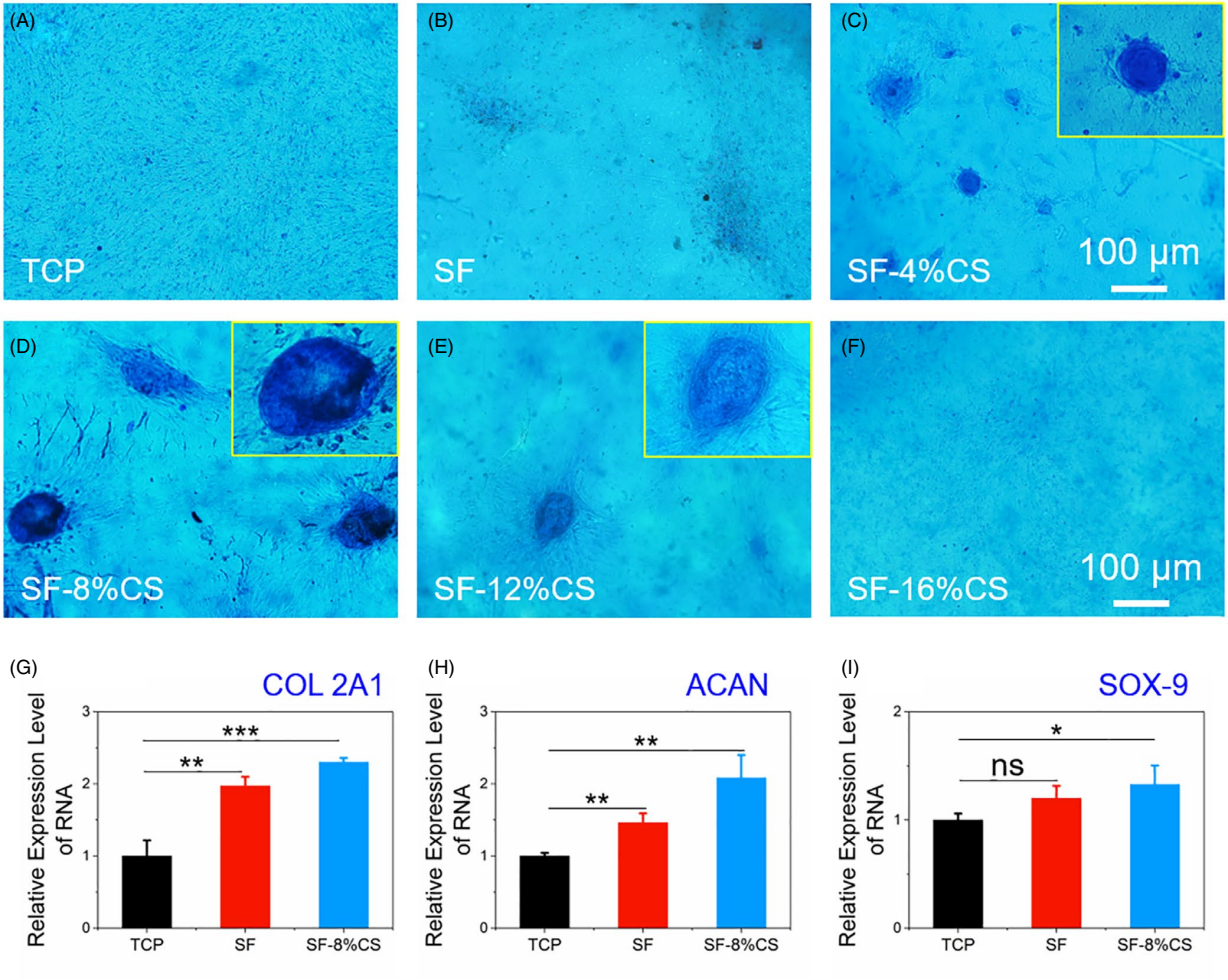
The chondrogenic differentiation of BMSCs on SF‐CS side. A‐F, Alcian blue staining of BMSCs cultivated on TCP and SF‐CS side with various concentrations of CS. G‐I, qRT‐PCR analysis of the relative mRNA levels of COL2A1, ACAN and SOX‐9 after cultured for 14 d. *P < .05, **P < .01 and ***P < .001, compared with TCP

The chondrogenic characterization of SF‐CS layer was analysed by Alcian blue staining and qRT‐PCR (Figure 4). BMSCs were cultured on SF side loaded with CS within CM for 14 days. By contrast, staining of cells on SF‐CS (8%) was more noticeable and presented highest glycosaminoglycan (GAG) content among all groups (Figure 4A‐F). SF films with higher CS concentrations (12% and 16%) presented lower GAG content, which should be attributed to the increased cytotoxicity. Therefore, 8% was chosen as the optimum concentration of CS for the following experiments. Furtherly, qRT‐PCR results proved that chondrogenesis‐related gene level of COL2A1, ACAN and SOX‐9 in SF‐CS (8%) group was 2.3‐, 2.1‐ and 1.3‐folds as much as that on TCP (*P* < .05, Figure 4G‐I). Interestingly, SF membrane partly promoted chondrogenic differentiation of BMSCs (COL2A1: 2‐folds, ACAN: 1.5‐folds and SOX‐9:1.2‐folds). Thus, SF and SF‐CS facilitated chondrogenic differentiation of BMSCs.

### 3.3 In vivo promotion effect on osteochondral defect repair

**Figure 5:**
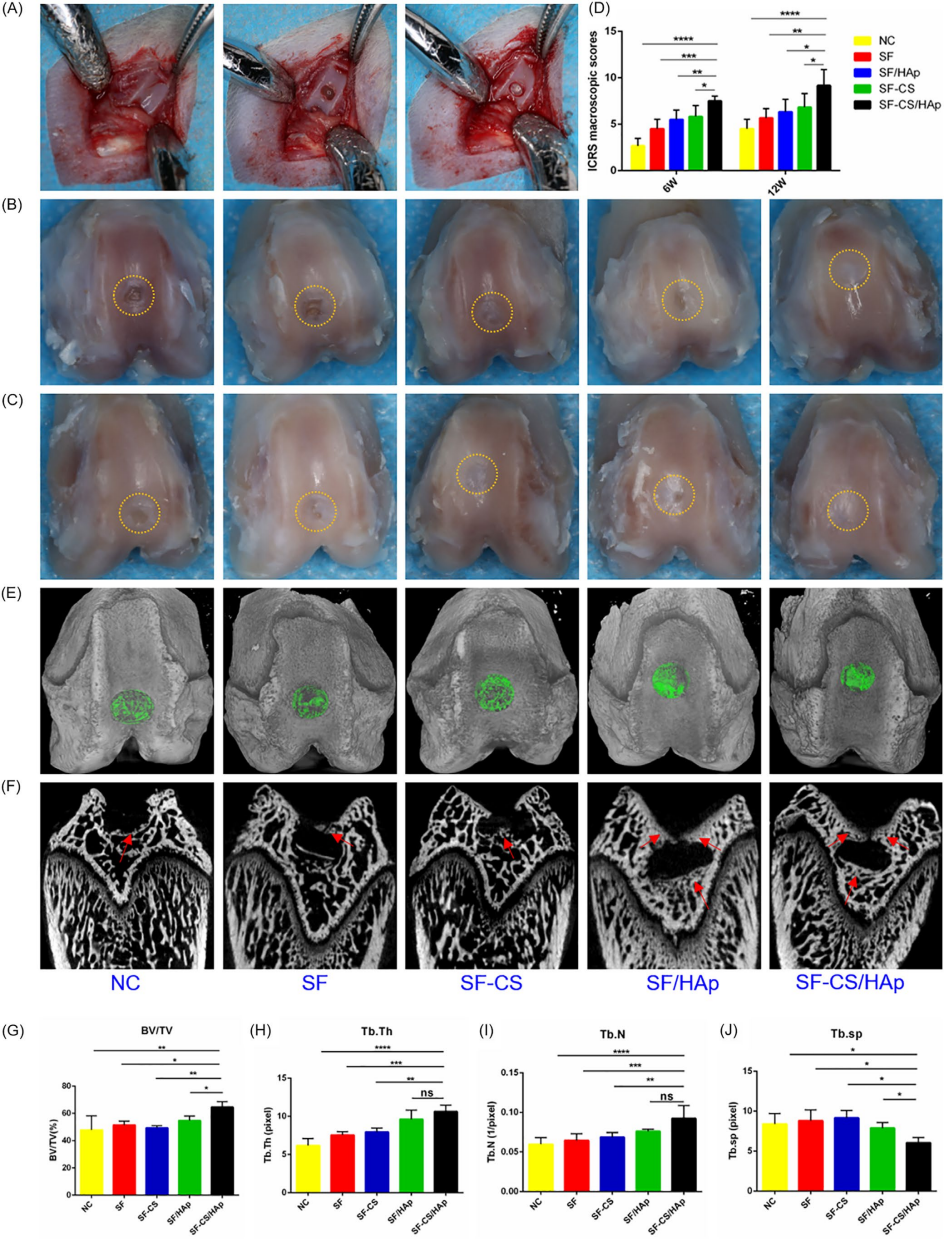
Gross specimen evaluation of repaired cartilage and micro‐CT evaluation of the subchondral bone regeneration in vivo. A, Photographs of femoral trochlear at the operation. B and C, Representative gross observation pictures of femoral trochlear defects at week 6 (B) and 12 (C) after surgery. The defect sites were marked with yellow circles. D, Macroscopic scores according to the ICRS macroscopic scoring system. E, The 3D digital reconstructed planform images. Green indicated the degree of bone defect healing. F, The 3D reconstructed transverse view images of different groups. Red arrows indicated the positions where more calcified tissues newly formed. G‐J, Quantitative analysis of the subchondral bone by reconstruction and analysis software. *P < .05, **P < .01, ***P < .001 and ****P < .0001 compared with SF‐CS/HAp group

Gross view, micro‐CT and histological analysis were used to confirm in vivo regenerative potential of SF‐CS/HAp membrane on osteochondral defect model. Figure 5A presented the general surgical procedure. At week 6 post‐surgery, the gross appearance of the cartilage defect was rough and the boundary between the newly formed tissues and the surrounding normal cartilage was obvious (Figure 5B). The defects in SF‐CS/HAp group were filled with newly formed tissues whereas only partial tissues covered the defect in NC, SF, SF/HAp and SF‐CS groups. At the 12th week after surgery, healing of the defect improved. In contrast to the other four groups, smooth and complete surface was found in SF‐CS/HAp group, and the newly formed tissues integrated commendably with the surrounding normal cartilage (Figure 5C). In accordance with International Cartilage Repair Society (ICRS) scoring standard, SF‐CS/HAp group had significantly higher macroscopic score than the other groups, suggesting the superiority of SF‐CS/HAp membrane in cartilage defect repair (Figure 5D).

The specimens of 12 weeks were scanned for subchondral bone regeneration analysis by micro‐CT. The 3D digital reconstructed images showed that SF‐CS/HAp group obtained a greater degree of bone defect healing than the other groups in spite of incomplete filling of subchondral bone in all groups (Figure 5E). In the transverse views of 3D images, the defects in SF/HAp and SF‐CS/HAp groups showed more newly formed calcified tissues in the bone‐occupied fractional area whereas fewer new bones were found in the defects of the other three groups (Figure 5F). The quantitative analysis of the reconstructed images was reflected by several indexes calculated from region of interest (ROI). The percentage of BV/TV significantly increased in SF‐CS/HAp group, which suggested that more new bones formed (Figure 5G). Tb.Th and Tb.N dramatically augmented, and Tb.Sp decreased as compared with the other groups, which implied that the newly formed bones were much denser in SF‐CS/HAp group (Figure 5H‐J). The SF‐CS/HAp membrane exhibited a better subchondral bone remodelling effect.

**Figure 6:**
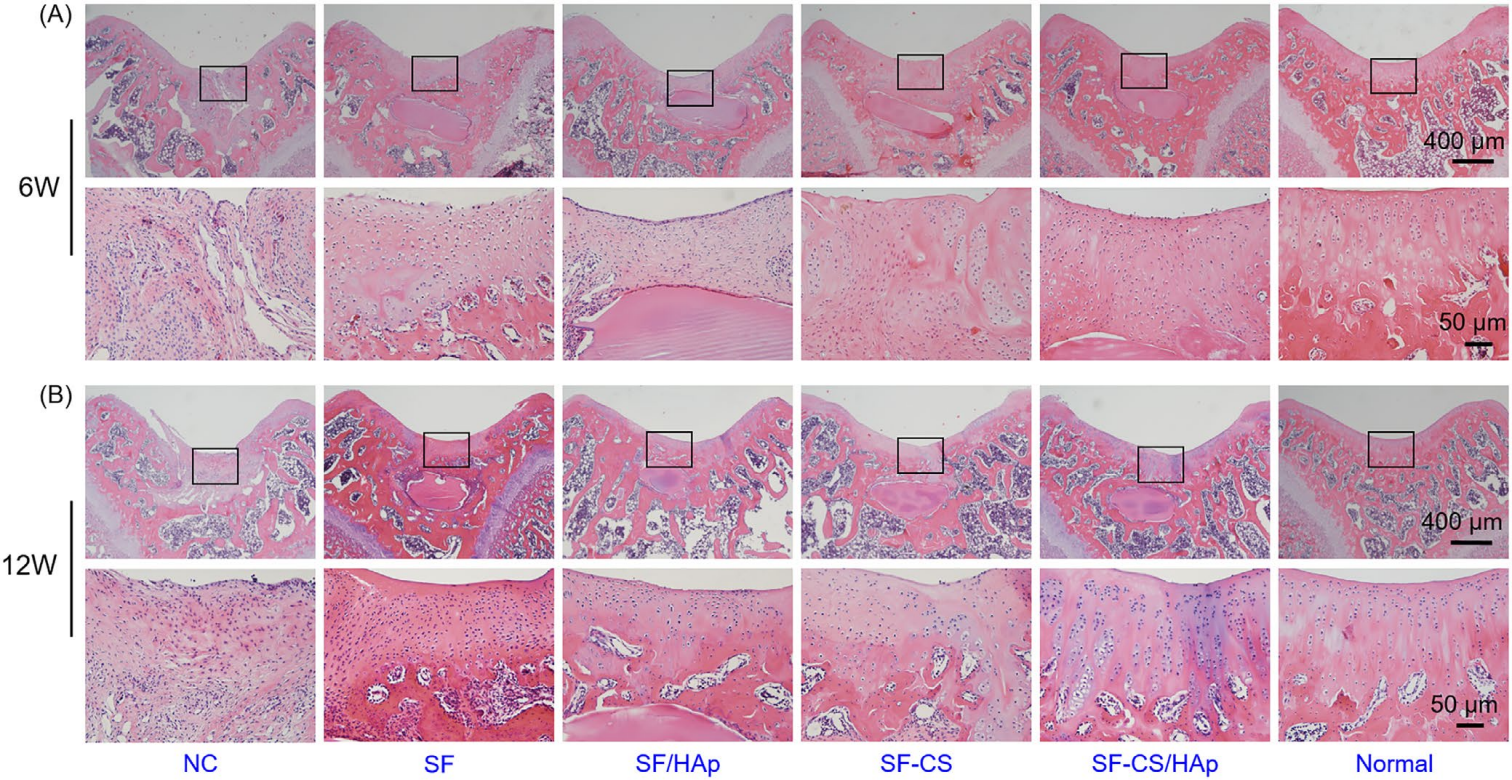
H&E staining images of the newly formed cartilage under different magnifications in different groups at week 6 (A) and 12 (B) after surgery. H&E staining images of normal tissues were also shown. The visual fields framed by the black box were magnified in the images below

Further, cartilage regenerative potential of the bilayer structure was revealed by histological analysis. H&E staining results were shown in Figure 6. At week 6 after surgery, the surface of the defects was irregular, and the boundary between the newly formed tissues and the surrounding normal tissues was distinct. Ingrowth of loose fibrous tissue with inflammatory cell infiltration could be seen in NC group, while a mixture of fibrous tissues and cartilage‐like tissues was apparent in the other groups. By contrast, the defects in SF‐CS/HAp group were mainly repaired by cartilage‐like tissues which possessed the characteristics of cartilage lacuna and agglomerate or single hyaline chondrocyte (Figure 6A). At week 12 after surgery, overall, the newly formed tissue was more mature in all groups in comparison with week 6. The defects were still filled with fibrous tissue in NC group whereas there was cartilaginous tissue in the other groups. In SF‐CS/HAp group, defects surface was smooth, and the boundary between newly formed cartilage and surrounding tissues disappeared. SF‐CS/HAp group had thicker cartilage layer and more obvious blue basophilic dyeing than other groups. Most importantly, the newly formed tissues had the most similar structure to the natural tissues and the optimal integration between the newly formed tissues and surrounding tissues (Figure 6B).

**Figure 7:**
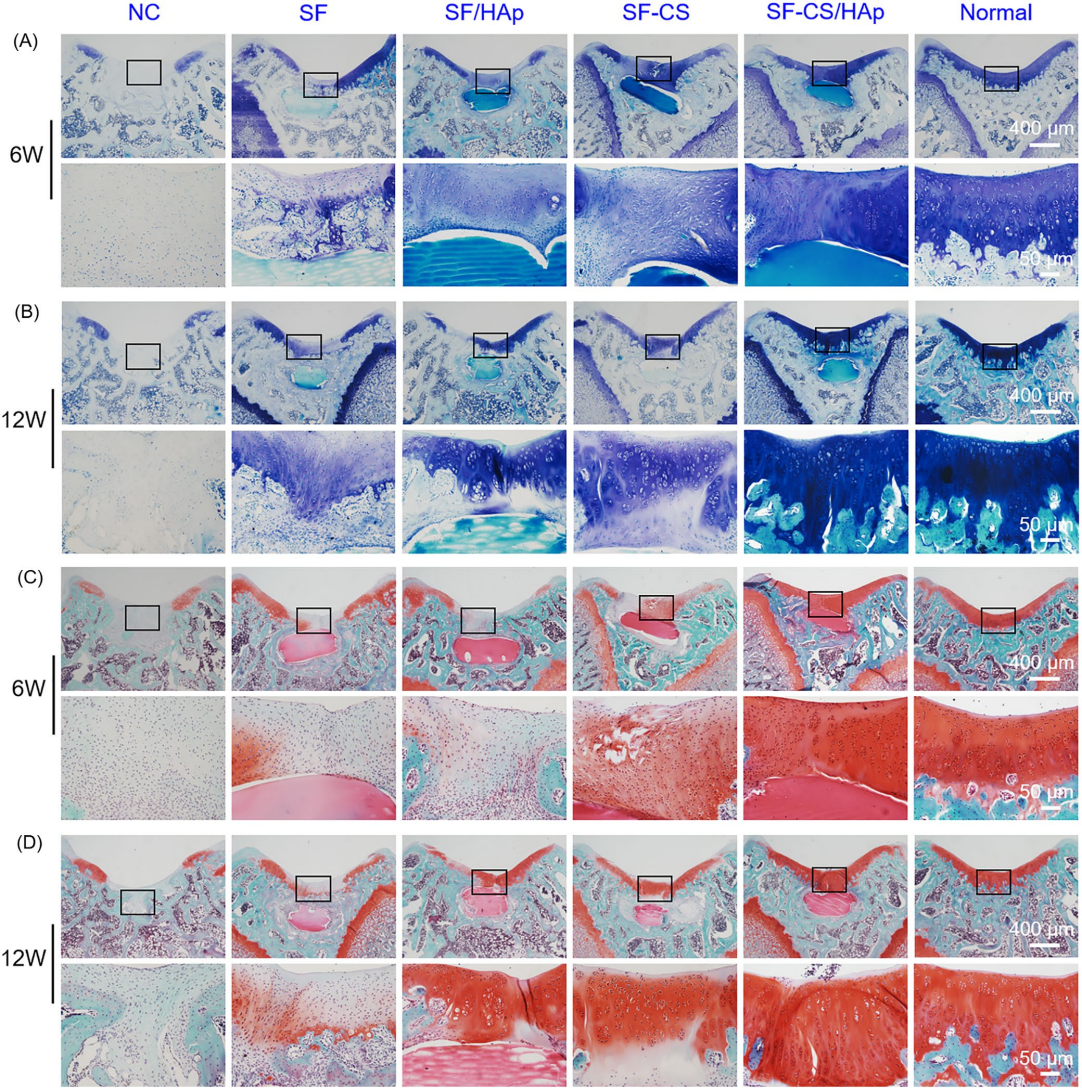
Toluidine blue staining (A,B) and Saf‐O staining (C,D) images of newly formed cartilage under different magnifications in different groups at week 6 and 12 after surgery. The staining images of normal tissues were also shown. The visual fields framed by the black box were magnified in the images below

The histological difference of new tissues was further confirmed by toluidine blue and Safranin‐O (Saf‐O) staining (Figure 7). The normal cartilage was stained into blue‐violet as positive staining. Six weeks after surgery, the cartilage layer staining was snatchy. The defects in SF‐CS/HAp group presented positive staining while NC group exhibited negative staining, and only faint staining in the other three groups (Figure 7A). Twelve weeks after surgery, NC group still showed negative staining, while positive staining was obvious in SF/HAp, SF‐CS and SF‐CS/HAp groups. SF‐CS/HAp group had more consecutive cartilage layers and more semblable compositions to natural cartilage (Figure 7B). The results of Saf‐O staining also provided compelling evidence for the cartilage regenerative capacity of the construct. Consistent with toluidine blue staining, only a thin layer of fibrous tissues was seen in NC group. Unsurprisingly, more regular cartilage layers (red staining) which had similar characteristics to the natural cartilage were formed in SF‐CS/HAp group, even though the three other experimental groups also obtained improved cartilage repair quality compared with NC group (Figure 7C,D). These results were further supported by the histological score for evaluation of cartilage repair at week 6 and 12, which showed that SF‐CS/HAp group acquired the highest scores among all groups (Figure S5).

**Figure 8:**
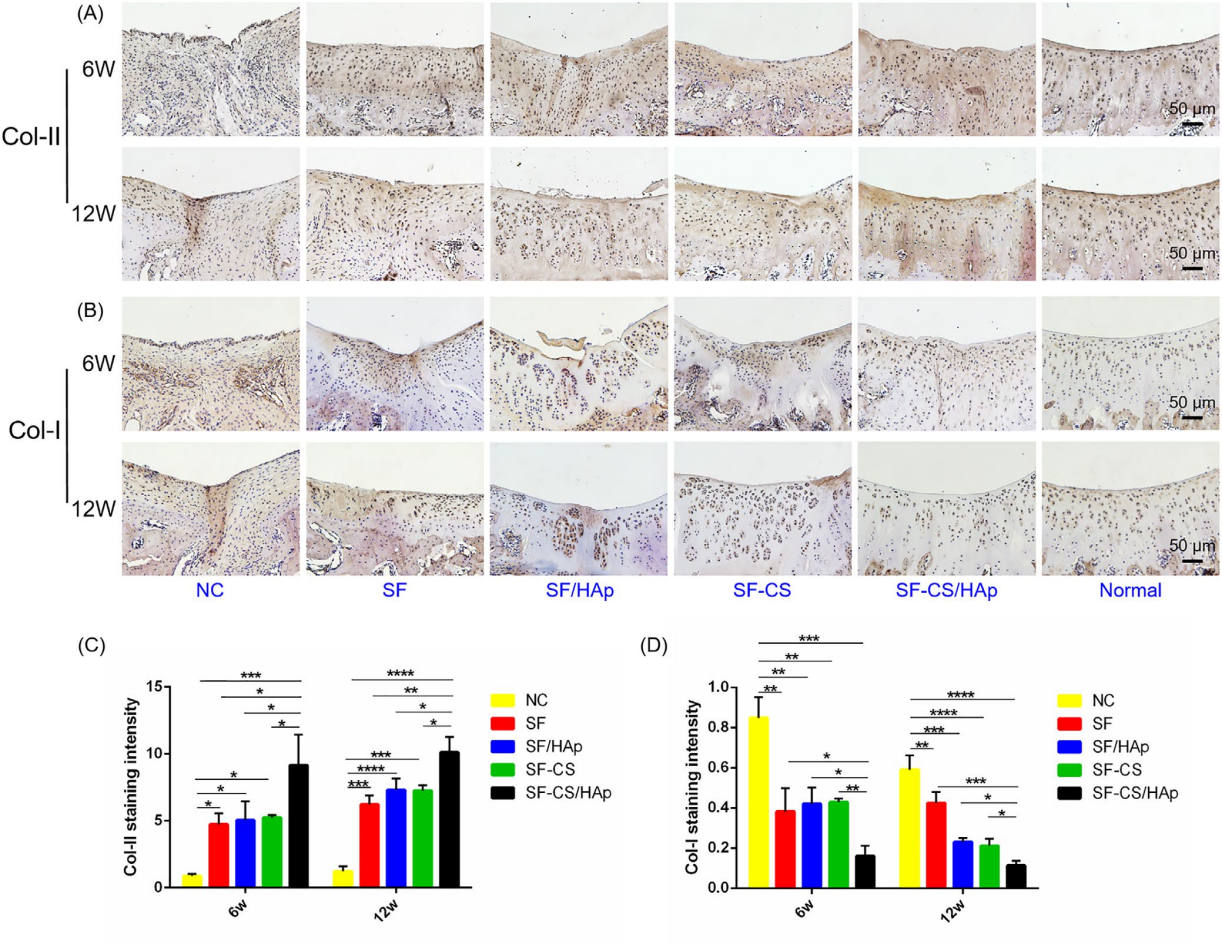
Immunohistological evaluation of newly formed cartilage by immunohistochemical staining of Col‐II (A) and Col‐I (B) images in different groups at week 6 and 12 after surgery. The immunohistochemical staining images of normal tissues were also shown. C and D, Quantitative analyses of Col‐II and Col‐I expression in five groups. *P < .05, **P < .01, ***P < .001 and ****P < .0001 compared with NC or SF‐CS/HAp group

To estimate collagens types in regenerated cartilage tissues, the expression of hyaline cartilage marker Col‐II, and fibrous cartilage marker Col‐I were evaluated by immunohistochemical staining (Figure 8). NC group contained abundant Col‐I indicating the formation of fibrous tissue or fibrous cartilage which cannot effectively act as a load bearing tissue in the articulations. In accordance with histological assessment, the newly formed tissues in SF‐CS/HAp group had higher expression of Col‐II (Figure 8A,C) and lower expression of Col‐I (Figure 8B,D) than the other groups, suggesting the regeneration of Col‐II‐enriched hyaline cartilage.

## 4 DISCUSSION

Concerns are growing about biomimetic restoration, and difunctional structure with concomitant chondrogenic/osteogenic potential draws increasing attention. In recent years, the concept of difunctional biomimetic repairing has been penetrated into the osteochondral tissue engineering scaffolds.^34^, ^35^, ^36^ In this study, bilayer SF‐CS/HAp membrane imitating the structure and function of osteochondral unit was developed to obtain osteochondral repairing, and the repairing ability was assessed in vitro and in vivo.

The bilayer structure was stratified into SF‐CS layer and HAp layer. The inseparable combination of the organic and inorganic phase was based on permeation of glutinous SF‐CS solution to HAp nanowires, and elements distribution mapping of cross‐section also presented the continuous and gradual transition region. The synthesized HAp short nanowires with length of several micrometres had typical morphology characteristics and crystal structures, which is identical to those in a previous study.^17^ The formation of bridged oxygen bond could integrate HAp short nanowires stably to form HAp nanotextured coating as HAp layer of SF‐CS/HAp membrane.^37^, ^38^ The intertwined linear structure of HAp short nanowires could provide an increasing friction between material and natural tissues when membranes were implanted in vivo. The irregular nanotextures on surface of the bilayer structure could provide beneficial physical stimulation to regulate cell differentiation.^30^, ^31^ Besides the typical peaks of HAp, XRD patterns of SF‐CS/HAp membrane exhibited a broad peak located at 2θ = 24°, corresponding to the crystalline diffraction of silk II with β‐sheet structure.^39^ β‐sheet structure could serve as the physical crosslinks to connect disparate protein chains to form continuous networks with high mechanical strength, avoiding complex chemical or physical treatments, and could also act as stress transfer centres to effectively adsorb energy and withstand deformation.^23^ Consistent with the XRD patterns, the typical peaks at 1513 and 1619 cm^−1^ in FTIR spectrum of SF‐CS/HAp membrane belonged to amide II and I vibration, respectively, which also meant the β sheets structure formation.^40^ In FTIR spectra for SF‐CS side of SF‐CS/HAp membrane, the peaks at 1224, 1063 and 923 cm^−1^ belonged to S=O or amide III stretching vibration, C–O–C stretching vibration of glycosidic linkage and ring vibration, respectively,^41^ which confirmed that CS was successfully incorporated into SF. The FTIR spectrum of SF‐CS/HAp membrane not only had the characteristic peaks of silk but also presented peaks of HAp and CS in respective sides, which illustrated that there was no change in the property of the three components.

Mechanical strength is one of the most critical factors for assessing the properties of cartilage repairing biomaterials, and sufficient mechanical strength is essential for bearing heavy loads and buffering stress.^2^ However, it is challenging to meet both bio‐properties and desired mechanical performance for biomimetic scaffolds in osteochondral repairing. In our study, tensile and compressive modulus (~5.26 and ~6.7 MPa) of SF‐CS/HAp membrane were in a reasonable range for the cartilage regeneration, and comparable to that of the natural cartilage tissue.^27^, ^28^ Ethanol‐induced β‐sheet structure was responsible for the favourable mechanical performances.^42^ In addition, SF could serve as a sustained release matrix for growth factors or protein drugs and preserve their potency successfully.^43^ The sustained release pattern of CS from SF‐CS/HAp membrane may be relevant to slowly biodegradable property of SF.^32^ As a naturally occurring protein polymer, SF possesses excellent biocompatibility, which is conducive to cell survival.^18^ CCK‐8 assay and Live/Dead cell staining proved that cell seeded onto SF membrane and SF‐CS (8%) membrane exhibited preferable cell viability. Regarding HAp nanowires, our previous study illuminated that the nanostructure of HAp short nanowires was in favour of cell survival and proliferation.^17^

The bio‐functionalization of developed biomaterials was conducive to further enhance its bioactivity. It is well known that surface nanostructures or decoration of bioactive motifs could regulate cells behaviours, and greatly improve the potential of cell differentiation, which is the key factor to the success of the implanted scaffolds.^17^, ^44^, ^45^ For example, a noncanonical Wnt5a motif could functionalize biomaterials in enhancing the osteogenesis and associated skeleton formation.^44^ After osteogenic induction, cells on HAp sides presented superior osteogenic differentiation potential as compared to SF sides and TCP, which may be due to the beneficial physical stimulation from the nanotextures of HAp surface.^30^, ^31^ In addition, the stiffness of HAp may also contribute to osteogenic differentiation.^46^, ^47^ Therefore, the nanotextured HAp layer has superior potential for bone regeneration. The chondrogenic differentiation ability, on the other hand, was preferable for cells on SF‐CS layer. Enhanced chondrogenic differentiation potential may be ascribed to CS releasing, moderate elasticity and surface nanotopography.^48^, ^49^ Consequently, we successfully fabricated the difunctional biomimetic SF‐CS/HAp membrane, wherein SF‐CS layer enhanced chondrogenic differentiation of BMSCs for cartilage regeneration, and HAp nanowire layer promoted osteogenic differentiation for subchondral bone restoration.

The histological evaluation by H&E, toluidine blue and Saf‐O staining in a rat osteochondral defect model showed that the four membranes acquired different levels of cartilage regeneration, whereas SF‐CS/HAp revealed the optimal chondrogenic effect among all groups. Furthermore, the structure and organization of cartilage's ECM are the primary determinants of normal function.^50^ Depending on the abundance, distribution and types of collagens and proteoglycans in cartilage's ECM, human cartilages are divided into hyaline cartilages, fibrocartilages and elastic cartilages. Articular cartilage is composed of hyaline cartilage which has a high density of Col‐II while fibrous cartilage, found mainly in the intervertebral discs, contains profuse Col‐I.^50^ SF‐CS/HAp membrane promoted the regeneration of Col‐II‐enriched hyaline cartilage thereby providing the function of physiologic loading in the joints. Equally importantly, SF‐CS/HAp group acquired better subchondral bone repairing than the other groups by micro‐CT analysis. Vascularized subchondral bone could supply nutrients and supporting for cartilage and thereby create a suitable surrounding for cartilage repairing.^51^ Potentially, the best cartilage regeneration capability of SF‐CS/HAp structure is not only ascribed to the chondrogenic induction from SF‐CS layer but also to the osteogenic promotion by HAp nanowire layer. Therefore, the bionic structure could serve dual functions for osteochondral regeneration.

**Scheme 1:**
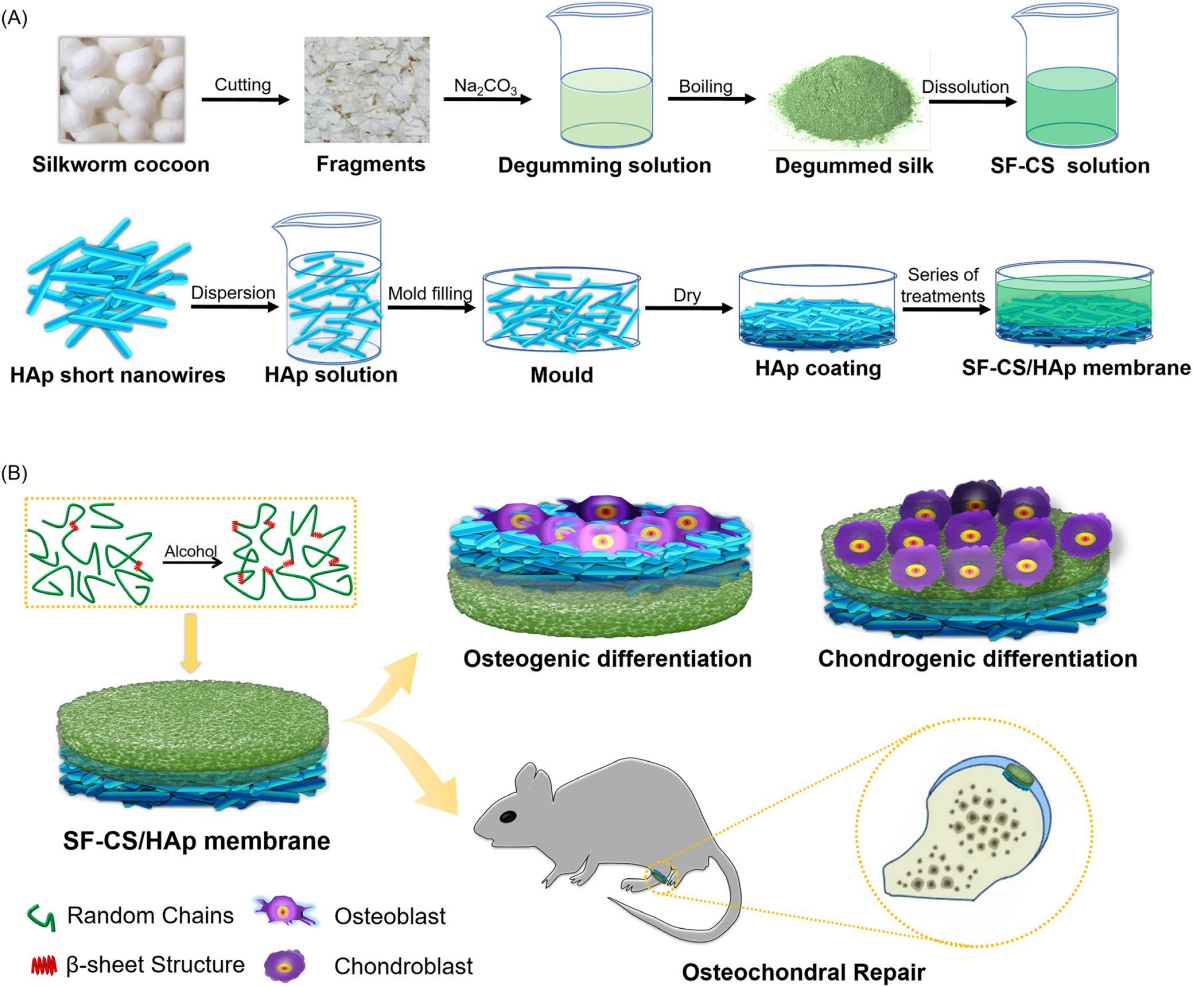
Schematics for construction of the bilayer biomimetic SF‐CS/HAp membrane for osteochondral repair. A, The preparation process of SF‐CS/HAp membrane. B, The schematic illustration of SF‐CS/HAp membrane for osteogenic/chondrogenic bi‐direction differentiation in vitro and osteochondral repair in vivo. SF was transformed into integral membrane by alcohol‐induced β‐sheet formation, which endowed SF with high toughness

In this study, we successfully developed a nanotextured SF‐CS/HAp membrane on the basis of biomimetic microstructure engineering design concept. The bilayer structure was stratified into SF‐CS layer and HAp nanowire layer, which was aimed to promote chondrogenic (SF‐CS layer)/osteogenic (HAp layer) bi‐direction differentiation in vitro and osteochondral repair in vivo (Scheme 1). The facile preparation process endowed SF‐CS/HAp structure with advantages of low cost and mass productive characteristics, which are conducive to further clinical application. The advantageous mechanical performance based on alcohol‐induced β‐sheet formation endowed SF‐CS/HAp structure with the immediate function of bearing and buffering. SF‐CS layer enhanced chondrogenic differentiation and nanotextured HAp nanowire promoted osteogenic differentiation of BMSCs. SF‐CS/HAp membrane had excellent osteochondral regeneration ability in vivo, and the newly formed cartilage‐like tissues had similar histomorphological characteristics to hyaline cartilage. Therefore, the biomimetic SF‐CS/HAp membrane with osteochondral repairing capacity could provide guidance for biomimetic tissue repair.

## CONFLICTS OF INTEREST

The authors declare that they have no conflicts of interest.

## AUTHOR CONTRIBUTIONS

LLS performed the experiments and wrote the manuscript. BJM performed the experiments and reviewed manuscript. FLW analysed the data and prepared figures. JHL, SS and XYL prepared figures, proofread and gave advice. HL and SHG designed the study and revised the manuscript.

## Supporting information

Fig S1‐S5

## Data Availability

The data that support the findings of this study are available from the corresponding author upon reasonable request.
