# Supplementary material for: Nanotextured silk fibroin/hydroxyapatite biomimetic bilayer tough structure regulated osteogenic/chondrogenic differentiation of mesenchymal stem cells for osteochondral repair
Source: Cell Prolif. 2020 Oct 1;53(11):e12917. doi: 10.1111/cpr.12917 (PMC7653257; doi:10.1111/cpr.12917)
Supplement: Supplementary file 1 — Fig S1‐S5 [file CPR-53-e12917-s001.docx]

**Supporting Information**

**Nanotextured Silk Fibroin/Hydroxyapatite Biomimetic Bilayer Tough Structure Regulated Osteogenic/Chondrogenic Differentiation of Mesenchymal Stem Cells for Osteochondral Repair**

Lingling Shang^1^, Baojin Ma^2^, Fulei Wang^2^, Jianhua Li^1^, Song Shen^1^, Xiaoyuan Li^1^, Hong Liu^2^, Shaohua Ge^2^

*1 Department of Periodontology, School and Hospital of Stomatology, Shandong University & Shandong Key Laboratory of Oral Tissue Regeneration & Shandong Engineering Laboratory for Dental Materials and Oral Tissue Regeneration, Jinan, Shandong 250012, China*

*2 State Key Laboratory of Crystal Materials, Shandong University, Jinan, Shandong 250013, China*

**Correspondence**

Hong Liu, No. 27 Shandanan Road, Jinan, China, E-mail address: hongliu@sdu.edu.cn

Shaohua Ge, No. 44-1 Wenhua Road West, Jinan, China, E-mail addresses: shaohuage@sdu.edu.cn

Lingling Shang and Baojin Ma contributed equally to this work and they should be regarded as co-first author.


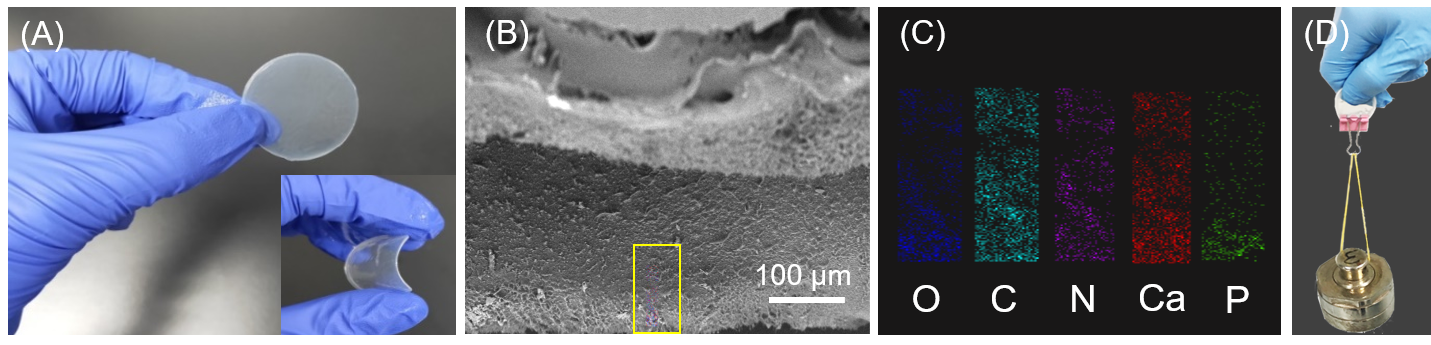


**FIGURE S1** The physical picture of SF membrane (A); SEM and the corresponding EDS mapping images of SF-CS/HAp membrane (B) and (C); (D) SF-CS/HAp membrane under tensile stress of the weight.


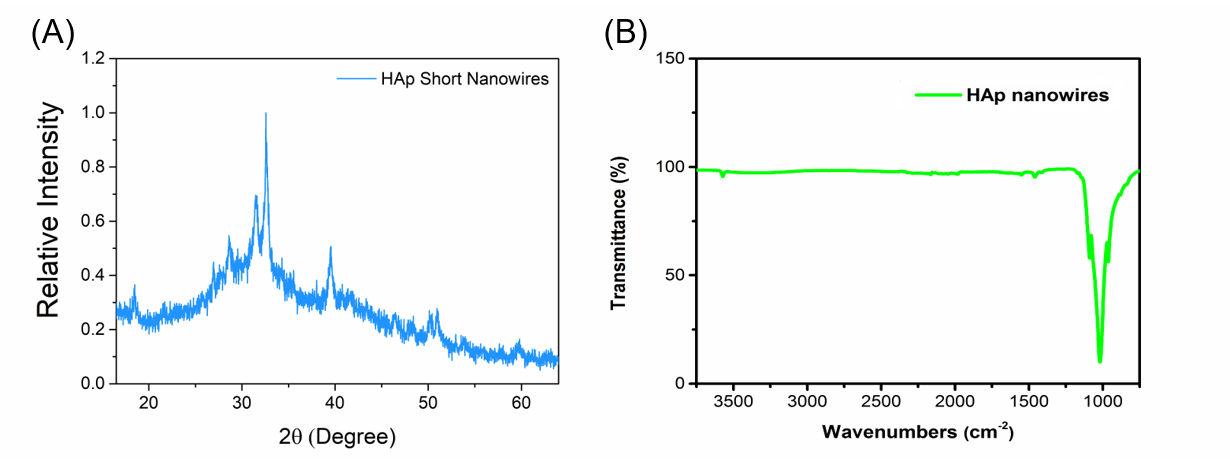


**FIGURE S2** The XRD pattern (A) and FTIR spectrum (B) of HAp short nanowires.

**

**

**FIGURE S3** Compression stress-strain curve of SF-CS/HAp membrane.





**FIGURE S4** Release curve of CS from SF-CS/HAp membrane.

**

**

**FIGURE S5** Histological scores for cartilage repair at week 6 and 12. ^*^*P*< .05, ^**^*P*< .01, ^***^*P*< .001 and ^****^*P*< .0001.
